# Supplementary material for: Magnetic Resonance Relaxometry for Tumor Cell Density Imaging for Glioma: An Exploratory Study via 11C-Methionine PET and Its Validation via Stereotactic Tissue Sampling
Source: Cancers (Basel). 2021 Aug 12;13(16):4067. doi: 10.3390/cancers13164067 (PMC8393497; doi:10.3390/cancers13164067)
Supplement: Supplementary file 1 [file cancers-13-04067-s001.zip › cancers_supplementary Figure S1-2.pdf]

cancers

MDPI

Supplementary Materials: Magnetic resonance relaxometry for tumor cell density imaging for glioma: An exploratory study via ¹¹C-methionine PET and its validation via stereotactic tissue sampling

Manabu Kinoshita, Masato Uchikoshi, Souichiro Tateishi, Shohei Miyazaki, Mio Sakai, Tomohiko Ozaki, Katsunori Asai, Yuya Fujita, Takahiro Matsuhashi, Yonehiro Kanemura, Eku Shimosegawa, Jun Hatazawa, Shin-ichi Nakatsuka, Haruhiko Kishima and Katsuyuki Nakanishi

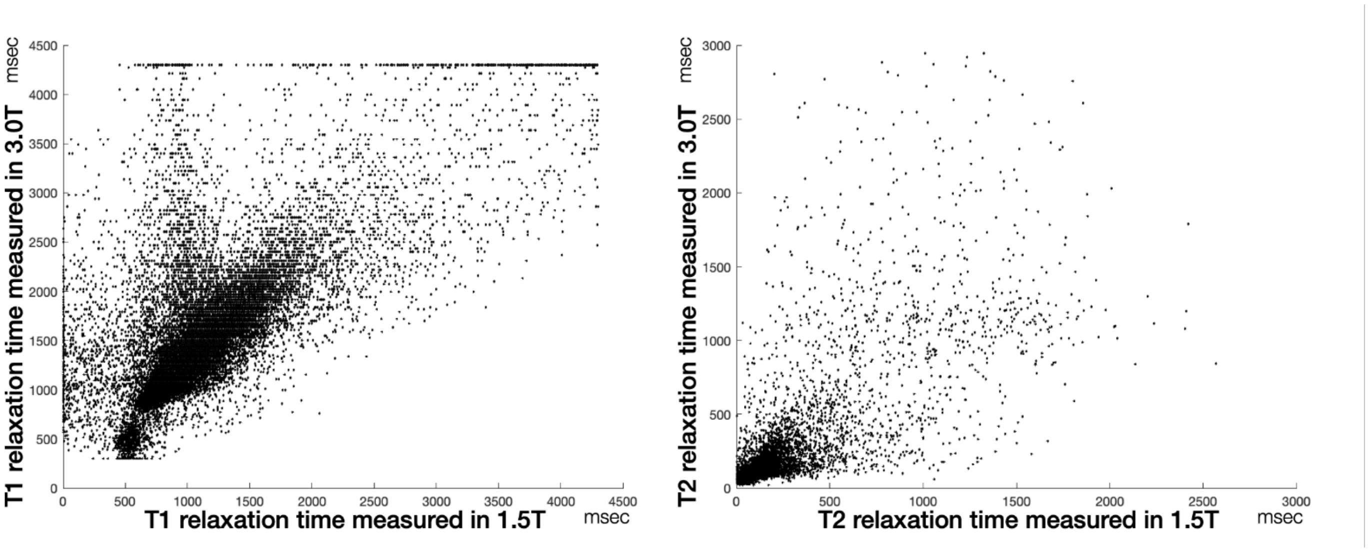

Figure S1. T1 or T2 relaxation times measured at 3.0T plotted as a function of those measured at 1.5T from a normal volunteer. Measured values are compared in a voxel-wise manner and the following conversion was obtained:.

$$T_{1@3T} = 1.19 \times T_{1@1.5T}, \quad \ldots (1)$$

$$T_{2@3T} = 0.92 \times T_{2@1.5T}, \quad \ldots (2)$$

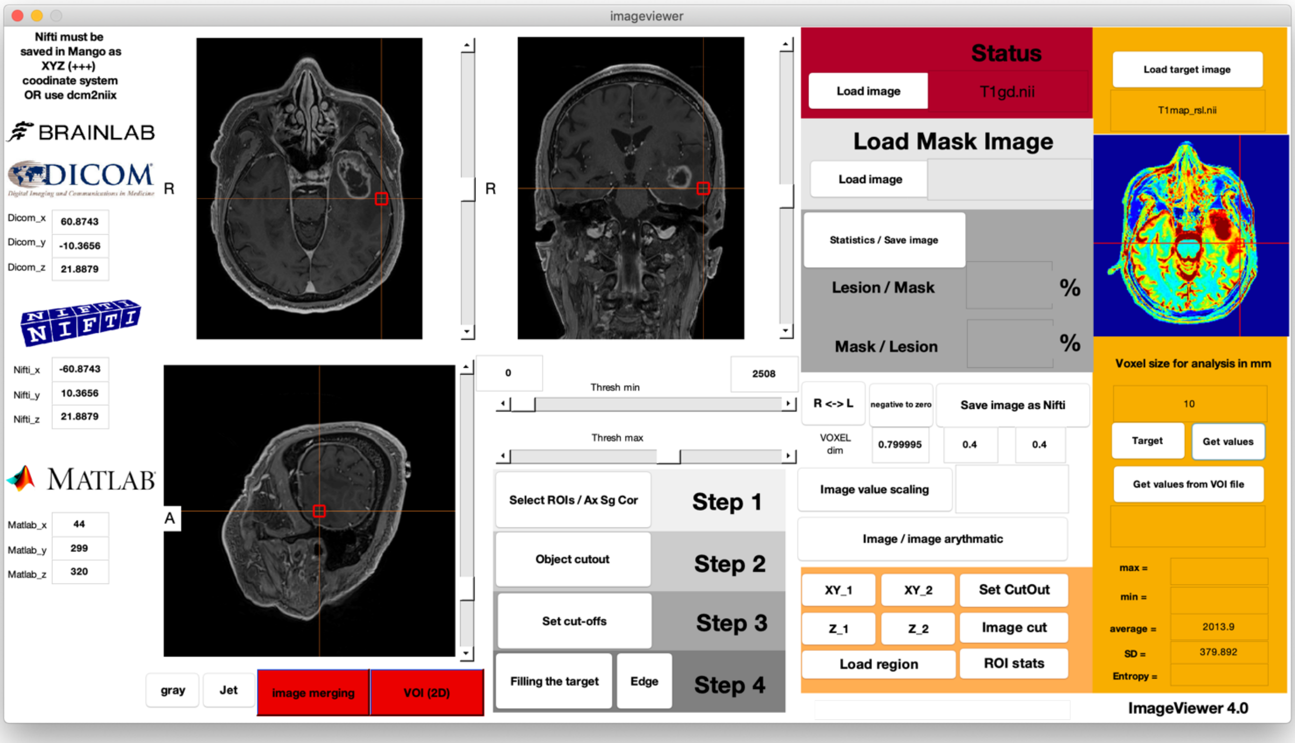

Figure S2. Software developed in-house was used to obtain measured values from images at locations of interest registered by the neuronavigation system, eliminating operator biases.
